# 

**Published:** 1899-05

**Authors:** 


### AMERICAN JOURNAL

OF

### DENTAL SCIENCE,

EDITED BY

F J S. GORGAS, M. D., D. D. S.

RICHARD GRADY, M. D, D. D. S.

VOLUME XXXIII—THIRD SERIES.

LIBRARY
SURGEON GENERAL'S OFFICE
FEB. 11-1901
168866.

BALTIMORE:
SNOWDEN & COWMAN MFG. CO.

LONDON:
TRUBNER & CO., PATERNOSTER ROW.

1900.

WI
AM45M

### Index to Vol. XXXIII Third Series.

A Few Considerations in Filling Teeth.................... 1
Arsenious Treatment for the Removal of Tumors from the Mouth ........................ 19
A Rubber Substitute From Corn.............................. 45
Asepsis........................................................ 47
A Low Fusible Metal........................................ 48
A Dictionary of Dental Science and Such Words and Phrases of the Collateral Sciences as Pertain to Art and Practice of Dentistry........................................ 90
A Case of Bronchitis and Pneumonia Caused by the Inhalation of the Filling from a [illegible] Broken in Extraction... 125
Antiseptic Uses of Oxygenated Waters...................... 140
A Decision Which May Cost Dentists Millions.............. 185
Alveolar Hemorrhage........................................ 229
A Peculiar Restoration .................................... 234
Alveolar Hemorrhage ....................................... 239
Arsenical Poisoning Cured by Orthoform.................... 240
A Mistake in Diagnosis..................................... 241
A Dentifrice................................................ 246
Amalgam Pills .............................................. 247
A Method of Pulp Canal Filling............................ 249
A New Method of Mummification of the Pulp................ 252
An Unusual Experience in Practice......................... 268
A Case of Tooth Impacted in the Left Bronchus: Gangrene of the Left Lung: Death ............................ 271
A Mouthful of Teeth ........................................ 281
A Laboratory Hint........................................... 283
Acidity of the Mouth During Sleep ........................ 288
An Alkalin Wash............................................ 335
A Plea for the Preservation of the Natural Teeth......... 358
A School Inspection........................................ 384
A Counter-Irritant ......................................... 384
A Case of Carcinoma of the Lower Jaw...................... 416
An Evil and Its Remedy .................................... 430
An Easy Method of Refining Gold........................... 462
Another Life Elixir.—French Scientist's Alleged Discovery of Secret of Youth ....................................... 494
A Remarkable Reflex........................................ 502
Amalgam Fillings in Teeth ................................. 527
An Evening With Bonwill.................................... 529
Arsenic in Oxyphosphates Not Injurious.................... 559

A New Alloy.... 570
A Young Licentiate of Quebec.... 573
Anchylosis of the Jaw Due to Interstitial Myositis.... 575
Broke a Tooth and Bled to Death.... 42
Bridge or Plate?.... 96
Bear This in Mind.... 143
Brittle Platinum Pins in Porcelain Teeth.... 289
Bacteriology in the Treatment of Disease.... 296
Broken Broach.... 336
Bleaching Devitalized Teeth With Pyrozone.... 410
Benjamin H. Catching, D. D. S.... 473
Concerning the Possibilities in the Art of Filling Teeth.... 49
Concerning Fees.... 86
Cinnamon Water as a Dressing.... 94
Circular Letter of the Foreign Relations Committee of the National Dental Faculties of America.... 133
Carbolic Acid Poisoning.... 142
Creosote and Sunlight.... 143
Cold Abscesses.... 143
Colorado State Dental Association.... 185
Combination Filling.... 231
Causes of High Palate.... 287
Cocainism.... 321
Combination Fillings.... 332
Cement.... 334
Compressed Cork and Its Uses.... 428
Clinical Demonstration of Dr. Bonwill's Methods of Practice. 504
College Athletics.... 523
Chloroform in India.... 572
Dentists in the Army.... 30
Dental Fees.... 48
Do Medical Colleges Strive to Thoroughly Educate Their Students?.... 84
Dr. Black's Theory.... 91
Dentifrice Soap.... 91
Does License to Practice Medicine Permit the Practice of Oral Surgery.... 93
Dentistry in the Army.... 236
Dental Jurisprudence.... 324
Dental Fees.... 422
Dental Science in 1480—As Expounded by Peter de Largelata.... 424
Dentistry in the Philippines.... 480
Dental Jurisprudence.... 481
Dr. B. H. Catching.... 520

Death From Cutting a Wisdom Tooth .... 526
Disease of the Maxillary Antrum Successfully Treated Through a Root Canal .... 556
Dentistry in France .... 572
"Dentistry" vs. "Mechanical Dentistry" .... 573
Eucaine .... 45
Extract from an Article upon "Some Overlooked Sources of Infection .... 83
Epilepsy .... 95
Extracting Superior Cuspids .... 141
Experiments with Nirvanin .... 191
Extraction of Teeth .... 383
Examining Boards Should be Controlled by State Societies. 567
Fatalities From Anesthetics .... 44
Filling Frail Teeth .... 47
Formaldehyde Gas .... 141
Failures in Bridge-Work .... 226
Facial Deformities .... 541
How to Remove a Pin Cemented to a Root .... 428
Hot Water Drinking .... 528
Inflammation of the Perodontal Membrane. Periodontitis, Alveola Abscess. Necrosis and Other Sequelæ .... 63
Is Dentistry a Remunerative Profession? .... 222
International Dental Congress .... 378
Interstate Comity in Dental Legislation .... 385
International Dental Congress .... 469
Is the Enamel a Vital Tissue? .... 526
Kentucky State Dental Association .... 472
Local Anesthetics .... 262
Legrand's Solution Anesthesique Hemostatique .... 576
Mercurial Necrosis Resulting from Amalgam Fillings .... 97
Method of Obtaining Perfect Metallic Dies for Gold or Aluminum Swaged Plates, Restoring all Undercuts as on Plaster Model .... 498
Microbes in the Arctic Regions .... 569
National Association of Dental Examiners .... 88
National Dental Association .... 88
Nervous Patients .... 175
National Association of Dental Faculties .... 193
Nervanin .... 300
New Method of Treatment of Wounds .... 431
Nervous Patients .... 447
Nitrous Oxid With Air or With Oxygen .... 539
On Restoring Consciousness .... 12
Opportunities for Success .... 38

Orthoform .... 142
Open Maxillary Sinus with Concomitant Maxillary Sinusitis. 150
Open Face Crown vs. Contour Filling or Richmond Crown for Incisors .... 265
Oil for Appendicitis .... 383
Oral Hygiene .... 460
Oxyphosphate Fillings Not Injurious .... 564
Porcelain Inlays .... 13
Pyorrhœa Alveolaris.—Personal Experience in Treatment.. 31
Pyorrhœa Alveolaris Treatment .... 44
Pyorrhœa Alveolaris .... 69
Pulp Mummification .... 145
Professional Demeanor .... 180
Pressure Anesthesia .... 209
Practical Results of Higher Education .... 217
Painless Extirpation of Live Pulps Without Cataphoresis.... 282
Painless Dentistry .... 376
Poor Man's Crown .... 426
Points in Favor of the Use of Alcohol and their Refutation.. 429
Paralysis Following General Anæsthesia .... 431
Personal Care of the Teeth .... 439
Plain Teeth With Plain Rubber .... 571
Removable vs. Fixed Bridge-Work .... 24
Repairing a Gold Crown .... 46
Reflex Dental Pain .... 257
Rest and Sleep .... 273
Refining Gold Scrap .... 285
Reminiscences of Dr. Bonwill .... 419
Root Filling .... 458
Resolutions on the Death of Dr. Bonwill .... 475
Report of the Pennsylvania Association of Dental Surgeons, 537
Repairing Rubber Plates .... 574
Real Diamonds .... 574
Some Notes on the Mixing of Amalgams .... 17
Silver Nitrate in the Treatment of Pulpless Teeth .... 35
Surgical Hints .... 39
Sensitive Dentine .... 45
Smokers' Teeth .... 46
Suggestions on Use of the Hypodermic Syringe .... 48
Supraorbital Neuralgia .... 92
Soldering Gold Crowns .... 93
Substitute for Rubber Gloves .... 96
Some Suggestions on the Treatment of Pyorrhœa Alveolaris. 119
Systemic Remedies in Diseases of the Teeth .... 169
Sterilization in Dental Practice .... 243

Sodium Salicylate for Toothache ........ 288
Suitable Recreations for Dentists ........ 309
Simple Test for Good Plaster ........ 332
Successful Amputation of Pulp ........ 33[illegible]
Steel Wire in the Mouth ........ 336
Some Mysterious Cases ........ 346
Solid Cast Gold Cusps for Crowns ........ 478
Sensitive Dentine ........ 572
Skulls and Brain Capacity ........ 574
Sterilizing Dental Instruments ........ 575
"Toothache" ........ 22
The Influence of Pregnancy on the Caries of the Teeth ........ 41
The Insertion of Dentures ........ 92
To Clean an Oil Stone ........ 92
The Deciduous Teeth and the Sixth Year Molars ........ 129
To Remove Gum Tissue from a Tooth Cavity ........ 143
Tannoform Cement ........ 143
To Replace a Broken Tooth ........ 144
Treatment of Carbolic Acid Poisoning ........ 192
To Apply the Rubber Dam ........ 240
The Deciduous Teeth—Their Uses and Diseases ........ 259
The Marvels of Surgery ........ 277
Teeth Made of Paper ........ 284
Treatment of Porcelain Facings to Prevent Fracture ........ 285
The Relation of Diseased Teeth to General Diseases ........ 286
The Influence of the Quality of Drinking Water in the Structure of the Teeth ........ 294
The Value of High Fusing Porcelain in Contour Work ........ 301
"The Outside of the Platter" ........ 329
To Dam a Molar ........ 336
The Niagara Meeting ........ 348
Three Vital Subjects ........ 368
The Wisconsin Decision ........ 372
Treatment of Abscess Swelling ........ 382
To Remove Gutta Percha Points from Root-Canals ........ 383
To Give a Fine Finish to Gold ........ 384
The Cause and Prevention of Dental Decay ........ 452
The International Tooth Crown Company vs. Kyle ........ 467
The Board of Dental Examiners of the State of Pennsylvania. 472
The Profession's Friends—The S. S. White Co., and the International Tooth Crown Co ........ 476
The Most Remarkable Clinic in the History of Dentistry ........ 509
The Wisconsin State Board of Dental Examiners ........ 514
The Kentucky State Dental Association ........ 519
The National Association of Dental Examiner ........ 519

The Brunette People of Europe........ 522
The Details of Constructing a Porcelain Bridge........ 549
The Sterilization of Water by Means of Ozone........ 570
The Useful Lemon........ 571
Under One Flag........ 381
Unusual Effect of Cocaine........ 479
Useful Hints for the Laboratory........ 496
Undercut Models........ 525
Varnishing Cavities........ 333
Vicarious Menstruation from the Gums........ 513
Where Pyrozone is Effective........ 46
Why Should New Jersey Compel Dentists to Register Annually........ 183
Will Fight the Decision........ 190
What are the Best Materials for Filling Children's Teeth.. 162
W. G. A. Bonwill Dead........ 331
"Waterproof" Cements........ 335
What Patients Should Know........ 337
What are the Best Materials for Filling Children's Teeth.... 433
Why the Better Element of the Dental Profession Does not Approve of Advertising........ 464
Zinc Dies........ 500

### To Readers and Correspondents.

Communications intended for publication, and exchange journals and papers, should be addressed to the Editor of The American Journal of Dental Science, No. 845 N. Eutaw St. Baltimore, Md. All letters relating to the business of the Journal, or containing cash remittances, to be directed to Snowden & Cowman Mfg. Co., 9 W. Fayette Street, Baltimore, Md. Articles for publication should be received by the Editor at least two weeks previous to the first of the month on which the number in which it is designed for them to appear is issued.

Broke a Tooth and Bled to Death ...... 42

Fatalities From Anesthetics ...... 44

Pyorrhœa Alveolaris Treatment ...... 44

A Rubber Substitute From Corn ...... 45

Sensitive Dentine ...... 45

Eucaine ...... 45

Repairing a Gold Crown ...... 46

Smokers' Teeth ...... 46

Where Pyrozone is Effective ...... 46

Asepsis ...... 47

Filling Frail Teeth ...... 47

Suggestions on Use of the Hypodermic Syringe ...... 48

A Low Fusible Metal ...... 48

Dental Fees ...... 48

☞ The American Journal of Dental Science will contain fifty pages of reading matter in each number, making a volume of about Six Hundred pages,

EXCLUSIVE OF ADVERTISEMENTS.

